# Practice pattern of ileal pouch surveillance in academic medical centers in the United States

**DOI:** 10.1093/gastro/gov063

**Published:** 2015-12-14

**Authors:** Jinyu Gu, Feza H. Remzi, Lei Lian, Bo Shen

**Affiliations:** ^1^Department of Colorectal Surgery, Digestive Disease Institute, Cleveland Clinic Foundation, Cleveland, OH, USA and; ^2^Center for Inflammatory Bowel Diseases, Digestive Disease Institute, Cleveland Clinic Foundation, Cleveland, OH, USA

**Keywords:** ileal pouch-anal anastomosis, neoplasia, restorative proctocolectomy, ulcerative colitis

## Abstract

**Objective**: There is no consensus on whether, when and how to surveil an ileal pouch. The aims of this study were to evaluate experts’ opinions and practice patterns on pouch surveillance and to determine if they were associated with detection of neoplasia.

**Methods**: Eligible physicians were identified by searching the literature in MEDLINE and the physician list of the Crohn’s and Colitis Foundation of America and surveying by questionnaire.

**Results**: Fifty-two eligible participants from 32 tertiary institutions were identified. Forty-one physicians (79%) felt that surveillance pouchoscopy was necessary, and 36 (69%) believed that pouchoscopy with biopsy was effective for the detection of neoplasia. Great variation exists with regard to the frequency of surveillance pouchoscopy. Eighteen physicians (35%) reported the detection of a total of 4 pouch dysplasias and 15 pouch cancers within the previous 5 years. The follow-up number of ileal pouches per year was significantly higher in the neoplasia detection group (50 *vs* 25, *P* = 0.041). Those who reported detecting neoplasia took even fewer biopsies from the ileal pouch body during the pouchoscopy examination (>3 biopsies per location, 44% *vs* 82%, *P* = 0.005). Multivariable analysis showed that the number of patients with ileal pouches followed up per year was the only independent factor associated with the detection of pouch neoplasia (odds ratio [OR]: 1.5; 95% confidence interval [CI]: 1.1–2.1; *P* = 0.005).

**Conclusion**: Most experts agree with performing pouchoscopy and biopsy for surveillance of ileal pouch neoplasia, although the optimal interval varies greatly. The detection of pouch neoplasia appears to be related to patient volume and physician experience.

## Introduction

Restorative proctocolectomy (RPC) with ileal pouch-anal anastomosis (IPAA) was developed by Parks and Nicholls in 1978. The procedure involves removal of diseased large bowel in patients with ulcerative colitis (UC) while preserving continuity of the gastrointestinal tract [1]. The procedure substantially reduces the risk for colitis-associated neoplasia (CAN). However, the surgery does not completely abolish the risk for CAN in patients with RPC and IPAA [2], especially in those with CAN detected by preoperative biopsy or diagnosed in specimens after colectomy [3, 4].

With refinement of the surgical technique, the majority of IPAA procedures are performed using a double-stapling technique without mucosectomy [5] that has raised the concern of neoplasm arising from the remaining rectal cuff or anal transitional zone (ATZ). However, even mucosectomy may not completely eliminate the mucosa [6, 7], and it has been shown that the procedure does not eradicate the risk for pouch neoplasia [3, 4].

There is no consensus on the need and interval of surveillance for neoplasia in patients with RPC and IPAA. One possible reason may be due to the low incidence of this complication with scant available data [8–11]. However, our previous study of 3 203 patients with RPC and IPAA for UC showed that 38 developed ileal pouch dysplasia or cancer during a mean 9.7 years of follow-up after the surgery [3]. Similarly, 25 cases of pouch neoplasia were identified in a nationwide cohort (from the Netherlands) of 1 200 patients with inﬂammatory bowel disease (IBD) and IPAA [4].

Considering the lack of standard surveillance guidelines for neoplasia after IPAA for UC, we speculated that the practice pattern of physicians is likely guided by personal experience and preference. A standard surveillance program, taking into account the individual risk factors, should be considered. The primary aim of this survey study was to assess the consensus on neoplasia surveillance using pouchoscopy among practicing clinicians and their practice patterns. Other aims of this study were to investigate the variations in the physicians’ opinions, practice patterns and experience on pouch surveillance and to evaluate whether they were associated with the detection of pouch neoplasia.

## Methods

### Inclusion and exclusion criteria

Gastroenterologists and colorectal surgeons potentially taking care of patients who have undergone RPC for UC or indeterminate colitis were identified by searching the literature on PubMed, Scopus, U.S. National Library of Medicine database (MEDLINE) and the physician member list of the Crohn’s and Colitis Foundation of America. The keywords used were as follows: “restorative proctocolectomy,” “ulcerative colitis,” “indeterminate colitis,” “carcinoma,” “adenocarcinoma” and “neoplasms.” The communication information from published papers (i.e. email address of the corresponding author) was used for contact.

Those physicians who responded and returned the questionnaire were considered to have consented to participate in this study. The questionnaire was re-sent twice in cases of no response or partially completed response. Incomplete questionnaires or physicians who indicated they did not routinely follow up IBD patients with ileal pouch were excluded from statistical analysis.

### Design of the questionnaire

The questionnaire, which requested basic information about the physician’s years in practice, experience in caring for IBD patients, knowledge of neoplasia after RPC and opinion on standard pouchoscopy surveillance, were sent by email using the Research Electronic Data Capture (REDCap) survey. The REDCap survey option being used was the anonymous survey form (Supplementary file).

#### Definition

Pouch-related neoplasia was defined as any dysplasia or cancer arising from the ileal pouch body or ATZ/rectal cuff in patients undergoing IPAA for UC, indeterminate colitis or Crohn’s colitis.

#### Statistical analysis

Univariable group comparisons were performed using Pearson’s chi-square test, Fisher’s exact test or Cochran-Armitage trend test to assess the associations between neoplasia detection and categorical variables. The relationship between neoplasia and continuous variables was evaluated using the *t* test or Wilcoxon rank sum test. A *P* value < 0.05 was considered statistically significant. Stepwise multivariable logistic regression analysis was performed, and ORs with 95% CIs were estimated. SAS 9.3 software (SAS Institute, Cary, NC) was used for all analyses.

## Results

A total of 118 physicians, including 86 gastroenterologists and 32 colorectal surgeons, was identified and surveyed by the questionnaire through REDcap. Of the 58 physicians (49%) who responded, six were excluded from analysis including one incomplete questionnaire and five physicians who reported that they do not routinely follow up IBD patients with ileal pouch. Altogether, 52 eligible participants (44%) from 32 tertiary institutions and their survey results were included. Six participants (11%) identified themselves as general gastroenterologists, 31 (60%) were gastroenterologists specializing in IBD, and 15 (29%) were colorectal surgeons. They had a mean of 16.1 ± 9.8 years of experience in providing IBD care and routinely followed a mean of 63 (median: 30, range: 6–1 200) patients with IPAA per year.

### Physician opinions

Forty-one physicians (79%) agreed that it is necessary to perform routine pouch surveillance for neoplasia arising from ileal pouch or ATZ/rectal cuff in all IBD patients undergoing IPAA. Forty-five physicians (87%) also agreed that patients who undergo mucosectomy may develop pouch neoplasia. Thirty-six physicians (69%) agreed that pouchoscopy with biopsy is effective for the detection of neoplasia. Twenty-two physicians (55%) believed that pouchoscopy solely for neoplasia surveillance should be performed every 2–3 years. Nine physicians (23%) believed that surveillance pouchoscopy should be performed annually. Seven physicians (18%) agreed about using an individualized protocol, and only two (5%) favored a 5-year plan.

### Practice patterns

The majority of the participants agreed about routine examination of the afferent limb (*n* = 49, 94%), ileal pouch (*n* = 50, 96%) and ATZ/rectal cuff (*n* = 51, 98%) during the pouchoscopy. Forty-eight (92%) and 46 (89%) physicians agreed with taking biopsies from the ileal pouch and ATZ/rectal cuff, respectively. However, only 24 (46%) and one (2%) physicians thought it was necessary to take biopsies from the afferent limb and perianal regions, respectively. Thirty-six (69%) physicians usually took >3 biopsies from the ileal pouch, while only 18 (36%) physicians took >3 biopsies from the ATZ/rectal cuff. Thirty-eight (73%) physicians thought bowel preparation with an oral agent was not necessary before pouchoscopy. The adult esophagogastroduodenoscope (EGD) (*n* = 32, 62%) was the most frequently used scope for the pouch examination.

### Factors associated with the detection of neoplasia

Altogether, 41 dysplasia and 15 cancers arising from the ileal pouch body or ATZ/rectal cuff were reported to have been found by 18 physicians (35%) within the prior 5 years. We then divided the participants into 2 groups: the neoplasia-reported group and the non-neoplasia-reported group. Univariable analysis demonstrated that the follow-up number of patients with IBD pouch per year was significantly higher in the neoplasia-detection group (50 *vs* 25, *P* = 0.041) (Table 1). Colorectal surgeons reported detecting more pouch neoplasia than both gastroenterologists specializing in IBD and general gastroenterologists (61% *vs.* 28% *vs* 11%, *P* < 0.001). There was no difference in years of physicians’ practice between the 2 groups. The physicians’ opinions on surveillance pouchoscopy between the 2 groups were also comparable (Table 2). There were no differences in locations of pouch inspection and biopsy during pouchocsopy examination between the 2 groups; however, those who reported neoplasia detection took fewer biopsies from the ileal pouch during the pouchoscopy examination (>3 biopsies per pouch anatomic location, 44% *vs* 82%, *P* = 0.005) (Table 3).

**Table 1.** Pouch neoplasia detection and physician practice pattern

	Physicians reporting neoplasia (*n* = 18)	Physicians not reporting neoplasia (*n* = 34)	*P value*
Number of pouch patients followed up per year	50 (6–1 200)	25 (10–40)	0.041
Field of practice, *n* (%)			<0.001
General GE	2 (11)	4 (12)	
GE specializing in IBD	5 (28)	26 (76)	
Colorectal surgery	11 (61)	4 (12)	
Years of practice	15.4 ± 8.1	16.4 ± 10.7	0.72
Number of IBD patients treated per month, *n* (%)			0.12
5–10	4 (12)	9 (17)	
10–50	11 (32)	19 (37)	
>50	19 (56)	24 (46)	

GE: gastroenterology

**Table 2.** Physician opinions on surveillance pouchoscopy

	Physicians reporting neoplasia (*n* = 18)	Physicians not reporting neoplasia (*n* = 34)	*P value*
Agree to perform routine pouch surveillance for neoplasia in IBD patients, *n* (%)	16 (89)	25 (74)	0.29
Frequency of performing routine pouchoscopy, *n* (%)			0.95
Every year	4 (25)	5(21)	
Every 2–3 years	8(50)	14(58)	
Every 5 years	1(6)	1(4)	
Individually	3(19)	4(17)	
Conditions necessitating pouch surveillance			0.62
Colitis-associated dysplasia/cancer	1 (50)	6 (67)	
Chronic pouchitis/cuffitis	0	2 (22)	
Other	1 (50)	1 (11)	
Agree that the risk of malignancy persists even after mucosectomy, *n* (%)	17 (94)	28 (82)	0.40
Agree that pouchoscopy with biopsy is effective for detecting pouch neoplasia, *n* (%)	15 (83)	21 (62)	0.11

**Table 3.** Practice patterns of physicians

	Physicians reporting neoplasia (*n* = 18)	Physicians not reporting neoplasia (*n* = 34)	*P value*
Location routinely observed during a pouchoscopy, *n* (%)			
Afferent limb	17 (94)	32 (94)	0.99
Ileal pouch body	17 (94)	33 (97)	0.99
ATZ or rectal cuff	18 (100)	33 (97)	0.99
Location routinely biopsied during a pouchoscopy, *n* (%)			
Afferent limb	7 (39)	17 (50)	0.44
Ileal pouch body	16 (89)	32 (94)	0.60
ATZ or rectal cuff	17 (94)	29 (85)	0.65
Perianal region	1 (6)	0	0.35
Number of biopsies usually taken from ileal pouch, *n* (%)			0.005
1–3	10 (56)	6(18)	
>3	8 (44)	28 (82)	
Number of biopsies usually taken from ATZ/rectal cuff, *n* (%)			0.77
1–3	12 (67)	20 (63)	
> 3	6 (33)	12 (38)	
Agree that bowel preparation is needed before pouchoscopy, *n* (%)	3 (17)	11 (32)	0.33
Scope usually used for pouchoscopy, *n* (%)			
Pediatric EGD	1 (6)	1 (3)	0.99
Pediatric colonoscope	5 (28)	6 (18)	0.48
Adult EGD	8 (44)	24 (71)	0.06
Adult colonoscope	4 (22)	3 (9)	0.22

The stepwise multivariable analysis showed that the number of IBD patients with ileal pouch followed up per year by a given clinician was the only independent factor associated with the detection of pouch neoplasia (OR = 1.5, 95% CI: 1.1–2.1, *P* = 0.005) (Table 4).

**Table 4.** Factors associated with the detection of pouch neoplasia

Factor	OR (95% CI)	*P value*
Number of IBD patients with ileal pouch followed up per year	1.5 (1.1–2.1)	0.005
Number of biopsies usually taken from ileal pouch	0.4 (0.1–1.8)	0.22
Agree that pouchoscopy with biopsy is effective for detecting pouch neoplasia	3.4 (0.5–23.1)	0.21
Agree that routine pouch surveillance is needed	79.3 (0.6–9926.9)	0.08

## Discussion

There is no published consensus or guidelines for endoscopy surveillance following RPC. Unlike surveillance protocol for pouch patients with underlying familial adenomatous polyposis (FAP) [12], standard protocol solely for the surveillance of neoplasia after IPAA for UC is still not available. It is well accepted that patients with dysplasia or adenocarcinoma in the original colectomy specimen are at higher risk of developing pouch neoplasia and require routine pouch surveillance [3,4,11]. However, whether surveillance endoscopy with biopsy in average-risk patients is necessary, together with questions of when and how, remains to be settled. Considering the ongoing debate on the topic, we carefully designed this survey study aiming to answer whether, how and when to do the surveillance. To our knowledge, this is so far the first study evaluating professional opinions and practice patterns on the surveillance plan for patients with ileal pouch after RPC for IBD disease. By assessing the varying opinions of practicing physicians about the need and frequency of surveillance pouchoscopy, the information from this study will provide valuable information for the development of a standard guideline.

Based on our data, 41 (79%) out of the 52 experienced gastroenterologists and colorectal surgeons from 32 US tertiary institutions have an aggressive stance about the surveillance for neoplasia arising from the ileal pouch body or ATZ/rectal cuff in all IBD patients undergoing IPAA. Moreover, the majority of the experts (87%) also believe that mucosectomy cannot completely eliminate the risk of pouch neoplasia. As to the approach of surveillance, 69% of physicians felt that pouchoscopy with biopsy was effective for the detection of neoplasia. The data indicate consensus among experienced physicians about the necessity and method of surveillance for pouch neoplasia after RPC, which warrants development of a standard surveillance program in the future. However, there are still >20% of physicians who disagree with this. One reason may be the low incidence of pouch-related neoplasia, which may discourage physicians from performing routine surveillance. Pouch neoplasia has been regarded as a rare complication, and routine surveillance is not advocated by some investigators [13–17]. Based on previous reports, the pooled cumulative incidence of pouch adenocarcinoma at 10 years after IPAA was 0.19% [11], and the pooled prevalence of confirmed dysplasia (after excluding “indefinite” for dysplasia) in the pouch and ATZ/rectal cuff was 1.13% [9]. However, the recent study of pouch neoplasia in a nationwide cohort of patients with IBD and IPAA reported that the respective cumulative incidences of pouch neoplasia were 2.0% at 10 years and 6.9% at 20 years [4]. This is consistent with the results of the largest study on this topic, which included 3 203 patients after RPC at our institution. The cumulative incidences of pouch neoplasia at 10 and 20 years were 1.3% and 4.2%, respectively [3]. The cumulative incidences of pouch neoplasia in recent reports seem much higher than the results from pooled data, implying that the actual incidence of pouch neoplasia might have been previously underestimated. In addition, more aggressive surveillance protocol may be needed in large referral centers where there is higher likelihood of detecting pouch neoplasia. Another factor that might affect the necessity of surveillance is the different techniques used in pouch procedures. Although a similar oncological outcome in patients with UC-associated dysplasia or cancer who underwent stapled or hand-sewn IPAA has been reported [18], it is believed that stapled IPAA may be associated with a higher risk of developing cancer from the anorectal mucosa. The reported risk of subsequent cancer arising from the residual anorectal mucosa increased 8 times if the stapled technique was employed [11], the possible reason being that even mucosectomy may not completely eliminate the mucosa [19, 20]. From pooled data, M'Koma *et al*. reported that 28 of 32 patients who developed pouch-related cancer in ATZ underwent mucosectomy [7]. Our data showed that the majority (87%) of the physicians believed that even mucosectomy is not sufficient to abolish the risk of pouch-related neoplasia. Considering the currently widespread use of the staple technique, it is reasonable to develop a standard plan for pouch neoplasia surveillance.

The optimal interval between the surveillance pouchoscopies is still controversial. As shown in our data, more than half of the physicians (55%) agreed that pouchoscopy should be performed every 2–3 years solely for the surveillance of neoplasia. An annual surveillance pouchoscopy plan was favored by 23% of the physicians surveyed, and 18% preferred an individualized plan. Only 2% of the physicians favored a 5-year surveillance protocol, reflecting their concern about the risk of pouch neoplasia. The diversity in physicians’ opinions might be attributed to the great difference in the volume of IBD patients with the ileal pouch (not FAP) who are routinely followed up every year (ranging from 6–1 200 patients).

As to surveillance method, approximately two-thirds of participants agreed that surveillance endoscopy with biopsy is effective for detecting pouch neoplasia. There were 11 physicians (21%) who felt that pouchoscopy with biopsy was not effective for the detection of neoplasia. Although pouch endoscopy with surveillance biopsy remains the gold standard for early detection and diagnosis of pouch neoplasia, the accuracy of early detection of dysplasia with endoscopy has been suboptimal [3,21]. Some pouch cancer has been detected without preceding endoscopic evidence of dysplasia, whereas other patients may not have endoscopically visible lesions even at advanced stages of pouch cancer. Therefore, endoscopy surveillance can still miss dysplasia or even cancer. New techniques such as DNA testing and imaging-enhanced endoscopy, which have better capabilities for detecting neoplasia, may be considered in the surveillance plan for high-risk ileal pouches [21,22]. Our data showed that more consensuses were found on the practice pattern (e.g. the location to be examined, location to be biopsied and the need for bowel preparation).

We then analyzed if physicians’ opinions and practice patterns were associated with the physician-reported neoplasia detection. To our knowledge, there are no reports on which factors are associated with the detection of neoplasia after IPAA for UC. Our data indicated that most of the aspects in physicians’ opinions and practice patterns were comparable between the two groups. Interestingly, univariable analysis showed that colorectal surgeons reported finding more pouch neoplasia than either gastroenterologists specializing in IBD or general gastroenterologists (61% *vs* 28% *vs* 11%, *P* < 0.001). The possible underlying explanation is that surgeons generally treat pouch cancers rather than gastroenterologists. The median follow-up number of IBD pouches per year was significantly higher in the neoplasia detection group (50 *vs* 25, *P* = 0.041), which by itself may increase the likelihood of detecting neoplasia. Another possibility of higher neoplasia detection might be attributed to the experience gained from the high volume of patient follow-up. We also found that those who reported successful detection of neoplasia took fewer biopsies from the ileal pouch during the pouchoscopy examination, implying that the physician’s experience significantly influenced the detection of pouch neoplasia. Multivariable analysis also demonstrated that the number of IBD-IPAA patients followed up per year is the only independent factor associated with the detection of ileal pouch neoplasia.

This study was limited by the small number of participants and selection bias. For confidential purposes, the demographic and institutional data of participating physicians were not included in the questionnaire, which made it impossible to determine if interesting variables such as gender and institutional preference influenced the opinion and practice pattern of the surveillance. Also, the results of neoplasia may be undermined by recall bias. There might have also been an issue of financial incentives for practicing physicians and/or their institutions when more procedures such as endoscopy were performed. However, we should cast no doubt upon professional integrity in the vast majority of health care professionals. The use of ancillary endoscopic imaging such as chromoendoscopy, narrow-band or ultrasound may also play an important role in the detection of neoplasia (which was not covered in the questionnaire). However, this is the first report specifically designed for this topic, which will lay the groundwork for future multicenter studies investigating the reasons for variations in practice and the subsequent ideal parameters that should be considered for standard surveillance protocols.

In conclusion, most experts agree with performing pouchoscopy and biopsy for ileal pouch neoplasia surveillance, although the optimal interval varies greatly. A standard surveillance protocol, taking into account the individual risk factors, should be considered instead. The detection of developing pouch neoplasia may be associated with the physician’s experience and patient volume.

## Supplementary Material

Supplementary Data
