# Supplementary material for: Practice pattern of ileal pouch surveillance in academic medical centers in the United States
Source: Gastroenterol Rep (Oxf). 2015 Dec 14;4(2):119–24. doi: 10.1093/gastro/gov063 (PMC4863190; doi:10.1093/gastro/gov063)
Supplement: Supplementary Data [file supp_4_2_119__index.html]

Supplementary Data 

# Practice pattern of ileal pouch surveillance in academic medical centers in the United States

## Supplementary Data

files

- Supplementary Data - pdf file
