# Supplementary material for: Practice pattern of ileal pouch surveillance in academic medical centers in the United States
Source: Gastroenterol Rep (Oxf). 2015 Dec 14;4(2):119–24. doi: 10.1093/gastro/gov063 (PMC4863190; doi:10.1093/gastro/gov063)
Supplement: Supplementary Data [file supp_gov063_Fig_1.pdf]

Practice of Surveillance Pouchoscopy for Neoplasia after Restorative Proctocolectomy for Patients with IBD Colitis

Please complete the survey below.

Thank you!

Your Name Please:

1. What is your field of practice?
   - ☐ General GI
   - ☐ GI specialized in IBD
   - ☐ Colorectal Surgeon
   - ☐ Other Specialty

   Other/Specify ______________________

2. How many years have you been practicing in this field since the completion of your training after residency ?
   ______________________ (years)

3. How many years have you been practicing in this field since the completion of your training after fellowship ?
   ______________________ (years)

4. How many IBD patients do you take care of per month?
   - ☐ < 5
   - ☐ 5-10
   - ☐ 10-50
   - ☐ >50

5. Do you have follow-up IBD patients with ileal pouch (EXCLUDING patients with familial adenomatous polyposis) every year?
   - ☐ No
   - ☐ Yes

6. Specify number IBD patients with ileal pouch (EXCLUDING patients with familial adenomatous polyposis) do you follow-up per year
   ____

7. Do you feel that it is necessary to perform routine pouch surveillance for neoplasia (dysplasia and/or cancer arising from ileal pouch or anal transitional zone/rectal cuff) in all IBD patients undergoing ileal pouch-anal anastomosis (IPAA)?
   - ☐ Yes
   - ☐ No

8. If "Yes", how often do you think it is needed to perform routine pouchoscopy SOLELY for the purpose of neoplasia in IBD patients with ileal pouch?
   - ☐ Every 1 Year
   - ☐ Every 2 -3 Years
   - ☐ Every 5 Years
   - ☐ Every 10 Years
   - ☐ Surveillance strategy should be individual, my plan is (if you would like to share)

8. If "No", under which condition would you consider pouch surveillance for neoplasia in IBD patients with IPAA?
   - ☐ Preoperative diagnosis of colitis-associated dysplasia/cancer in colon or rectum
   - ☐ Long lasting colitis (history > 10 yrs after diagnosis)
   - ☐ History of primary sclerosing cholangitis
   - ☐ Family history of colon cancer
   - ☐ Chronic pouchitis and/or cuffitis during follow-up
   - ☐ Other

   Other/Specify ______________________

9. Do you believe that patients who undergo mucosectomy (excision of the rectal mucosa) may also develop pouch neoplasia?
   - ☐ Yes
   - ☐ No

   If "Yes", what is your surveillance plan for those patients?
   - ☐ Every 1 Year
   - ☐ Every 2 -3 Years
   - ☐ Every 5 Years
   - ☐ Every 10 Years

10 Do you feel pouchoscopy with biopsy is effective for the detection of dysplasia/cancer?
   - ☐ Yes
   - ☐ No

11. Which part(s) of the pouch do you routinely observe during a pouchoscopy?
   - ☐ Afferent limb (neo terminal ileum)
   - ☐ Pouch per se (inlet, outlet, and body of the pouch)
   - ☐ Anal transitional zone (ATZ) or rectal cuff

   (Multiple Answers)

12 Which part of the pouch are biopsies usually taken during a pouchoscopy?
   - ☐ Afferent limb (neo terminal ileum)
   - ☐ Pouch per se (inlet, outlet, and body of the pouch)
   - ☐ Anal transitional zone (ATZ) or rectal cuff
   - ☐ Perianal region

   (Multiple Answers)

13. How many biopsies do you usually take from ileal pouch during the pouchoscopy examination?
   - ☐ 1-3
   - ☐ >3

14. How many biopsies do you usually take from anal transitionalzone/rectal cuff during a pouchoscopy examination?
   - ☐ 1-3
   - ☐ > 3

15. How many cases of dysplasia arising from ileal pouch or anal transitionalzone/rectal cuff have you found within the past 5 years?

   ____

16. How many cases of cancer arising from ileal pouch or anal transitionalzone/rectal cuff have you found within the past 5 years?

   ____

17. Do you think bowel preparation with an ORAL agent is needed before pouchoscopy?
   - ☐ Yes
   - ☐ No

18. Which scope do you usually use in pouchoscopy examination?
   - ☐ Pediatric esophagogastroduodenoscope
   - ☐ Pediatric colonoscope
   - ☐ Adult esophagogastroduodenoscope
   - ☐ Adult colonoscope

Do you have any comments or suggestions for this survey?

____
